# Expression of renin–angiotensin system components in the early bovine embryo

**DOI:** 10.1530/EC-12-0013

**Published:** 2012-06-21

**Authors:** Wioletta Pijacka, Morag G Hunter, Fiona Broughton Pipkin, Martin R Luck

**Affiliations:** 1 School of Biosciences University of Nottingham Sutton Bonington Campus, Loughborough, Leicestershire, LE12 5RD UK; 2 New Maternity Unit Nottingham University Hospitals NHS Trust City Hospital Campus, Hucknall Road, Nottingham, NG5 1PB UK

**Keywords:** angiotensin II, embryo, development, receptor

## Abstract

The renin–angiotensin system (RAS), mainly associated with the regulation of blood pressure, has been recently investigated in female reproductive organs and the developing foetus. Angiotensin II (Ang II) influences oviductal gamete movements and foetal development, but there is no information about RAS in the early embryo. The aim of this study was to determine whether RAS components are present in the pre-implantation embryo, to determine how early they are expressed and to investigate their putative role at this stage of development. Bovine embryos produced *in vitro* were used for analysis of RAS transcripts (RT-PCR) and localisation of the receptors AGTR1 and AGTR2 (immunofluorescent labelling). We also investigated the effects of Ang II, Olmesartan (AGTR1 antagonist) and PD123319 (AGTR2 antagonist) on oocyte cleavage, embryo expansion and hatching. Pre-implanted embryos possessed AGTR1 and AGTR2 but not the other RAS components. Both receptors were present in the trophectoderm and in the inner cell mass of the blastocyst. AGTR1 was mainly localised in granular-like structures in the cytoplasm, suggesting its internalisation into clathrin-coated vesicles, and AGTR2 was found mainly in the nuclear membrane and in the mitotic spindle of dividing trophoblastic cells. Treating embryos with PD123319 increased the proportion of hatched embryos compared with the control. These results, the first on RAS in the early embryo, suggest that the pre-implanted embryo responds to Ang II from the mother rather than from the embryo itself. This may be a route by which the maternal RAS influences blastocyst hatching and early embryonic development.

## Introduction

The renin–angiotensin system (RAS) is mainly associated with the regulation of blood pressure and ion homeostasis. The classically described circulating RAS comprises angiotensinogen (AGT) produced in the liver, renin (REN) produced by the juxtaglomerular cells of the afferent renal arteriole and angiotensin II (Ang II) generated by angiotensin converting enzyme 1 (ACE), an exopeptidase produced in vascular endothelial cells (1, 2, 3). The RAS complex operates through interactions between several proteins and peptides. AGT, an α2-globulin, is cleaved by REN to form the decapeptide Ang I (4). Two amino acids from the carboxyl terminus of this peptide are removed by the membrane-bound ACE to form Ang II. Ang II interacts mainly with two cell membrane receptors, type 1 (AGTR1) and type 2 (AGTR2). In general, mammalian AGTR1s are predominant during adult life and AGTR2s are predominant in foetal life (5, 6).

Components of the RAS have been found in the oviduct, in the placenta and in the foetal membranes of various species including bovine (7, 8). In the bovine oviduct, Ang II stimulated by LH and ovarian steroids regulates the peak of oviductal contractions, helping gamete transport to the fertilisation site (9). Bovine placenta and foetal membranes contain both of the best-described Ang II receptors. The AGTR1 and AGTR2 are co-located in foetal and maternal tissues but appear at different densities. The maternal side of the placenta mainly possesses AGTR1. On the foetal side, AGTR2 is predominant: it is found in the allantochorionic membrane of the placentomes (cotyledon), in the mesenchymal cells on the foetal side of the cotyledon and in the foetal villi. The density of AGTR2 decreases at the end of gestation similar to that of AGTR1. The high density of AGTR1 at the beginning of gestation is associated with higher content in maternal tissue in early placentomes (10).

Several studies have shown that RAS is active during early foetal development of many species, and a high concentration of Ang II receptors, especially AGTR2, has been reported in mouse, rat and human foetuses (11, 12, 13, 14). Analysis of foetal RAS has been performed mainly in rodents. It was shown that Ang II binds mostly in the rat sub-epidermal skin layer, in mesenchymal and connective tissues, in skeletal muscle, and also in kidney, liver, blood vessels and gastrointestinal tract (15). AGTR1 occurs in the kidney, adrenal cortex and aorta in both foetal (19 day) and adult rats (11). The earliest reported immunodetection of Ang II receptors in the mammal (rat foetus) was on day 10, by which time most organogenesis is complete (11).

Physiological studies on embryo development have shown that Ang II plays the role of a growth factor, particularly in the post-implantation rat embryo (16). Culture of embryos with a physiological concentration of Ang II (10^−11^ M) significantly increased somite number and the number of branchial bars and also improved mandibular, optic and otic development. To date, nothing is known about Ang II's role in the pre-implantation embryo or generally about RAS involvement in early embryonic development.

The aim of this study was to determine whether RAS components are present in the pre-implantation embryo, to determine how early they are expressed and to investigate their putative role at this stage of development. Bovine embryos produced *in vitro* were used for the expression and location of RAS components in the embryo from the two-cell stage until day 19 of development. Cultured embryos were treated with Ang II, together with AGTR1 and AGTR2 antagonists, to investigate the effect of the hormone and its receptors on pre-implantation embryo development.

## Materials and methods

All reagents were supplied by Sigma Aldrich Co. Ltd. unless otherwise stated.

### Transcript analysis

Total RNA from bovine embryos was extracted and DNase I treatment was performed using Absolutely RNA Nanoprep Kit (Stratagene, Cambridge, UK). Bovine kidney and liver total RNA were used as controls and extracted using Microprep Kit (Stratagene). RT was performed on RNA from 20 embryos or 4 μg of control tissue RNA using an AffinityScript Multiple Temperature cDNA Synthesis Kit (Stratagene). β-Actin (*ACTB*) was used as a housekeeping gene. RT-PCR was performed using Taq DNA Polymerase with Standard Taq Buffer (New England BioLabs, Hitchin, Herts, UK) in an iCycler thermocycler (Bio-Rad) according to the manufacturer's specifications. Primers for PCR amplification of the bovine-targeted genes *AGTR1, AGTR2* and *AGT* were designed using the online program Primer3 (http://frodo.wi.mit.edu/cgi-bin/primer3/primer3_www.cgi). Primer sequences for β-actin were obtained from (17) and those for *ACE* and *REN* were kindly made available by Dr Peter Masters (QMC, Nottingham, UK). All primers crossed an exon:exon boundary to eliminate the risk of amplifying genomic DNA, except for *AGTR1* and *AGTR2* that have a coding region localised in one exon: primers for those two genes were designed in this region.

For each set of primers, a PCR was performed on kidney or liver cDNA. Products were confirmed by sequencing at the Biopolymer Synthesis and Analysis Unit, University of Nottingham. Briefly, PCRs were carried out with the RNA equivalent of two embryos and the cDNA was diluted 1:10 when *ACTB* was run. Negative controls with PCR mix only were also performed. The number of PCR cycles was 44 for *ACTB* and 50 for all RAS components (Table 1). Twenty microlitres of each PCR product were run on a 1.8% agarose gel containing 0.5 μg/ml ethidium bromide and analysed by GelDoc-It Imaging System (Upland, CA, USA).

### Immunofluorescent labelling of hatched blastocysts

Hatched blastocysts were fixed in 2% paraformaldehyde (PFA)/0.2% Triton X-100 for 30 min at room temperature. Blastocysts were then washed in 1× PBS/0.1% Tween 20 washing solution and transferred to 5% BSA/1× PBS per 0.1% Tween 20 blocking solution for overnight incubation at 4 °C. The following day, the bovine blastocysts were transferred to primary antibody diluted in blocking solution as follows: 1:200 AGTR1 (ab47162) or 1:200 AGTR2 (ab19134; Abcam, Cambridge, UK) for overnight incubation at 4 °C. This was followed by washing and a 1 h incubation with goat anti-rabbit biotinylated secondary antibody (1:200 dilution; Vector Laboratories, Inc., Peterborough, UK) (18). Subsequently, blastocysts were washed in a washing solution and incubated with 1:200 streptavidin/FITC (Invitrogen) for 1 h in the dark at room temperature. Embryos were washed again before mounting in DAPI (Vector Laboratories, Inc.). To enhance the blue signal from nuclei, they were incubated for 15 min with 10 μg/ml Hoechst DNA stain (H6024; Sigma). Slides were covered with a coverslip, sealed and examined under a fluorescent microscope by Simple PCI (Hamamatsu Corporation, Ann Arbor, MI, USA). Overall, 26 embryos were analysed for AGTR1 localisation, 25 for AGTR2 and 28 for control. The specificity of AGTR1 and AGTR2 antibodies was demonstrated by western blotting using blocking peptides: ab91523 and ab91522 (Abcam). Kidney was used as a positive control.

### Oocyte collection and *in vitro* maturation

Bovine ovaries were obtained from a local abattoir and transported to the laboratory in thermal containers containing 1× PBS at 39 °C. Cumulus–oocyte complexes were aspirated from 2 to 10 mm follicles and oocytes were selected according to a four-point scale based on the number of compact cumulus cell layers and granulation of the oocyte cytoplasm, as described previously (19). Groups of 50–70 oocytes were matured in bicarbonate-buffered TCM 199 medium with supplements (1.36 mM glutamine, 10 μg/ml porcine LH (AFP12389A, NHPP, NIDDK, Torrance, CA, USA), 10 μg/ml porcine FSH (Vetropharm, Belleville, ON, Canada), NIH-FSH-P1 1 mg/ml 17β-oestradiol, 0.5% penicillin/streptomycin and 10% FCS; pH 7.3–7.4; 270–290 mOsmol) in four-well dishes (Nunclon, Roskilde, Denmark), incubated for 20–24 h at 39 °C, 5% CO_2_ in humidified air (type BB 6220 CU; Heraeus, Hanau, Germany).

### Sperm preparation, IVF and *in vitro* culture

Spermatozoa from a single bull in cryopreserved straws (Supersires, Devon, UK) were thawed, transferred to 15 ml polystyrene conical tube and overlaid with 3 ml Ca^2^^+^-free medium. Tubes were kept at an angle of 60° in the incubator for 1 h to allow swim up. Subsequently, the supernatant containing the live sperm was centrifuged at 800 ***g*** for 10 min. The supernatant was discarded, leaving about 0.1–0.2 ml media above the pellet level, and this was topped up with 0.8–0.9 ml fertilisation medium (93.08 mM NaCl, 3.08 mM KCl, 0.22 mM Na_2_HPO_4_·2H_2_O, 1.52 mM MgCl_2_·6H_2_O, 26.18 mM NaHCO_3_, 5.3 mM CaCl_2_·2H_2_O, 0.00014 U heparin, 0.2 μM epinephrine, 1.39 mM caffeine, 0.4 μM hypotaurine, 0.5% penicillin/streptomycin, 9.87 mM sodium pyruvate, 0.6% BSA (fatty acid free), 3.7 ml/l sodium lactate (60% syrup); pH 7.7; 270–290 mOsmol), in which the spermatozoa underwent capacitation. Drops of 500 μl of the final sperm suspension were prepared in four-well dishes, which were kept in the humidified incubator until the oocytes were prepared (20).

Matured oocytes were selected according to cumulus expansion and the appearance of the cytoplasm. Oocytes with an expanded cumulus and an even cytoplasm with no granules were partially denuded in oocyte washing medium (116 mM NaCl, 5.90 mM KCl, 0.22 mM Na_2_HPO_4_·2H_2_O, 1.52 mM MgCl_2_·6H_2_O, 5.3 mM CaCl_2_·2H_2_O, 19.90 mM NaHCO_3_, 20 mM Hepes, 0.5% penicillin/streptomycin, 1.86 ml/l sodium pyruvate, 0.6% BSA (fatty acid free), 6.6 mM sodium lactate (60% syrup); pH 7.3–7.4; 270–290 mOsmol) so that only three to five layers of cumulus remained attached to the oocyte. Fifty oocytes were incubated for 24 h in 500 μl fertilisation medium containing 1×10^6^ motile spermatozoa/1 ml and then completely denuded. Putative zygotes were cultured in 500 μl synthetic oviduct fluid (SOF) medium supplemented with 0.4% BSA (SOF–BSA) in four-well dishes. On day 3, cleaved embryos were transferred from SOF–BSA into 500 μl SOF medium supplemented with 10% FCS (SOF–FCS) and cultured at 39 °C in 5% CO_2_, 10% O_2_ and 85% N_2_ in a humidified atmosphere (20). The culture medium was changed every 48 h. All *in vitro*-produced embryos used in these studies (for transcripts, protein analyses and functional studies) were generated by the same protocol.

Embryos were generated *in vivo* as follows. Multiparous, lactating Holstein–Friesian cows, maintained within the University of Nottingham commercial herd, were inseminated at naturally occurring oestrus (day 0) by Genus technicians (Genus PLC, Westmere Drive, Crewe, UK) using semen from beef bulls of proven fertility. On days 12, 14, 16 and 18, cows were transported to an adjacent abattoir and the uterus was collected immediately following killing (by captive bolt and exsanguination) and transported to the laboratory. The uterine horns were dissected free from surrounding tissues and separated past the bifurcation. The horn ipsilateral to the corpus luteum was then flushed with 20 ml saline into a petri dish and the embryo, if present, collected and stored at −80 °C for subsequent analysis.

### Effects of Ang II and AGTR1 and AGTR2 antagonists on oocyte cleavage, embryo expansion and hatching

Bovine embryos produced *in vitro* were used to test the effects of Ang II (A9525; Sigma Aldrich Co. Ltd.) at two concentrations (10^−11^ and 10^−9^ M) on fertilisation, expansion and hatching. Experiments were run in two batches at different times of the year. Similarly, the effects of 10^−6^ M Olmesartan (5-methyl-2-oxo-2H-1,3-dioxol-4-yl)methyl4-(2-hydroxypropan-2-yl)-2-propyl-1-((4-[2-(2H-1,2,3,4-tetrazol-5-yl)phenyl]phenyl)methyl)-1H-imidazole-5-carboxylate; kindly provided by Daiichi Sankyo, Germany) and 10^−6^ M PD123319 (Sigma Aldrich Co. Ltd.; AGTR1 and AGTR2 antagonists respectively) were investigated. Olmesartan was dissolved in 10^−3^ M NaOH. The concentration of Ang II in the experiments with antagonists was 10^−9^ M. Treatment was applied each day from fertilisation until collection.

### Statistical analysis

Data were analysed using SPSS Version 17 (Chicago, IL, USA). The proportions of cleaved oocytes and expanded and hatched embryos were analysed by ANOVA; if data were significant, the analysis was followed by Bonferroni's *post hoc* test. Differences of *P*<0.05 were considered significant. All data are presented as a mean±s.e.m.

## Results

### Transcript analysis

*In vitro*-produced embryos were collected at 2-, 4-, 8-, and 16-cell, expanded and hatched blastocyst stages. Embryos recovered *in vivo* were at 12, 14, 16 and 19 days of development.

**Figure 1:**
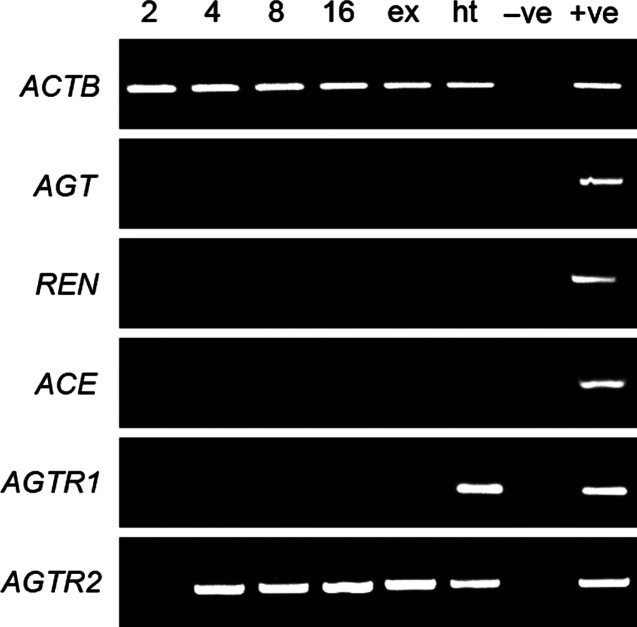
Transcript expression for renin–angiotensin system components in bovine embryos at 2-, 4-, 8- and 16-cell stage; ex, expanded blastocyst; ht, hatched blastocyst; −ve, negative control; +ve, positive control.

**Figure 2:**
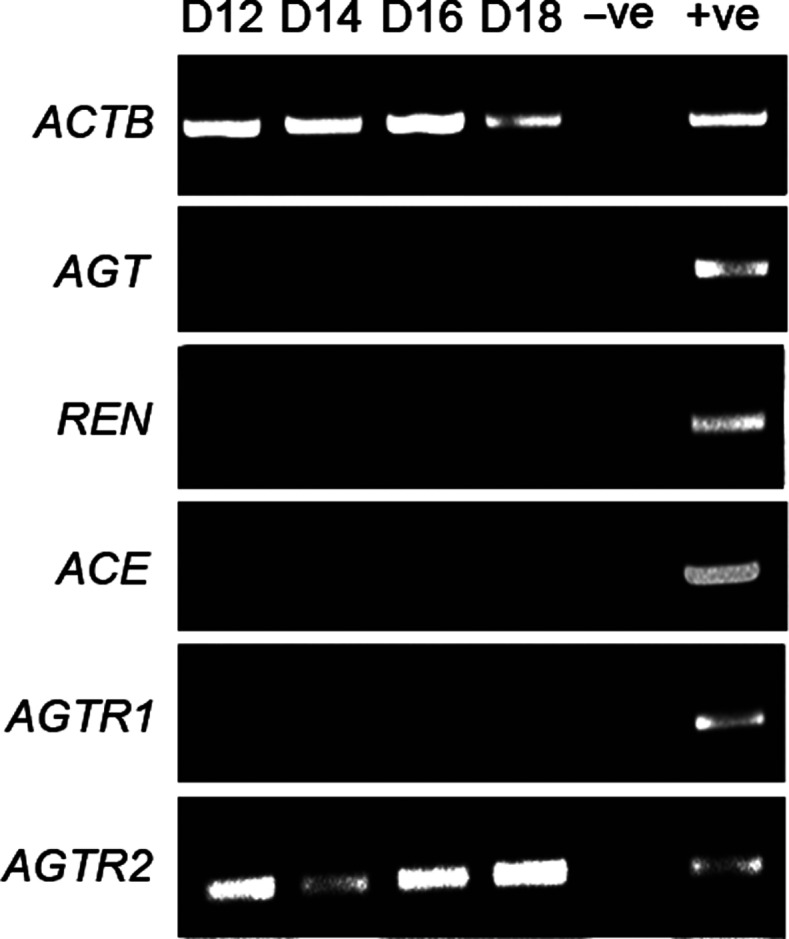
Transcript expression for renin–angiotensin system components in elongated trophoectoderm from bovine embryo: D12, day 12; D14, day 14; D16, day 16; D18, day 18 of development; −ve, negative control; +ve, positive control.

The components of RAS (*ACE, REN, AGT, AGTR1* and *AGTR2*) were analysed by RT-PCR in bovine embryos produced *in vitro* from five independent experiments and in the trophectoderm from two experiments. The *AGTR2* amplicons were present in cleaved oocytes, in all 4-, 8- and 16-cell stage embryos and in expanded and hatched blastocysts. They were also detected in trophectoderm recovered from embryos at days 12, 14, 16 and 19 of development *in vivo*. A very weak signal for *AGTR1* was present in one of the five samples of expanded blastocyst and in two of the five samples of hatched blastocyst. Transcripts of *ACE, REN* and *AGT* were not detected in any analysed samples (Figs 1 and 2).

### Immunolocalisation

**Figure 3:**
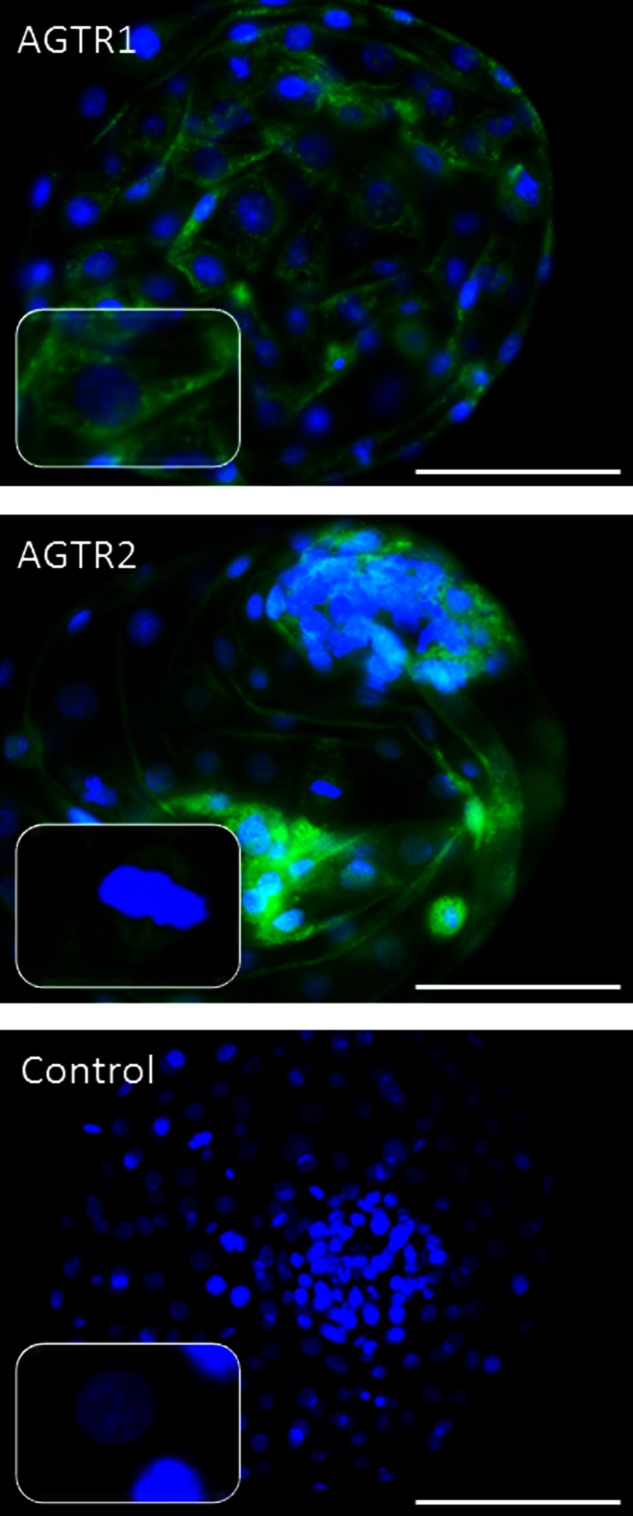
Immunolocalisation of AGTR1 and AGTR2 in day 8 bovine hatched blastocyst produced *in vitro*. A, AGTR1; B, AGTR2; C, control (secondary antibody only). Pictures show merged images of nucleus stained with DAPI (blue) and FITC signal from the localised protein (green). Scale bar is 100 μm. In the left bottom corner of each photograph, magnification of a single cell has been included.

Immunofluorescent labelling located the AGTR1 and AGTR2 in whole mounted, day 8, hatched blastocysts. AGTR1 was present in the trophectoderm and in the inner cell mass of the blastocyst. It showed typical membrane localisation, although the staining was very weak. In contrast, a very strong signal was observed in the granular-like structures in the cytoplasm, suggesting the existence of clathrin-coated vesicles following ATG1R internalisation (Fig. 3). The AGTR2 was found mainly in the nuclear membrane with very intense staining in the mitotic spindle of trophoblastic dividing cells (Fig. 3). AGTR2 was also present in the cell cytoplasm of the inner cell mass and trophectoderm.

### Effects of Ang II and inhibitors on embryo development *in vitro*

**Figure 4:**
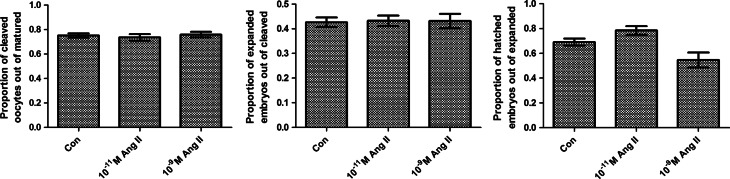
Effect of Ang II on oocyte cleavage and expansion and hatching of embryos cultured *in vitro*. There was no significant effect of 10^−11^ M Ang II and 10^−9^ M Ang II (*P*>0.05). All the values are presented as mean±s.e.m., *n*=10 cultures.

Approximately 120 oocytes were used per treatment in each of ten replicate experiments. There was no significant influence of Ang II treatment, at either of the concentrations tested, on the proportions of cleaved oocytes, expanded and hatched embryos (*P*>0.05; Fig. 4).

**Figure 5:**
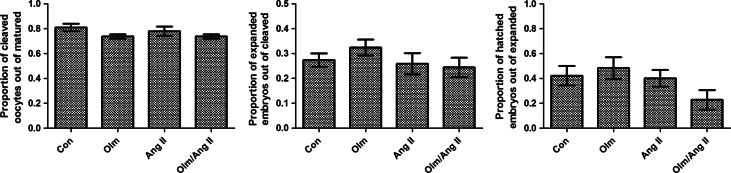
Effect of Olmesartan on oocyte cleavage and expansion and hatching of embryos cultured *in vitro*. No effect of treatment was observed (*P*>0.05). All values are presented as mean±s.e.m., *n*=7 cultures.

Treatment with Olmesartan was used to look at the effect of AGTR1 blockade on embryo development. Approximately 50 oocytes were used in each treatment or control group, in each of seven experimental replicates. The inhibitor was dissolved in 10^−3^ M NaOH before addition to the culture medium, the solvent being shown to have no effect on embryo development (*P*>0.05). Olmesartan (10^−6^ M) had no effect on oocyte cleavage, embryo expansion or hatching (*P*>0.05; Fig. 5).

**Figure 6:**
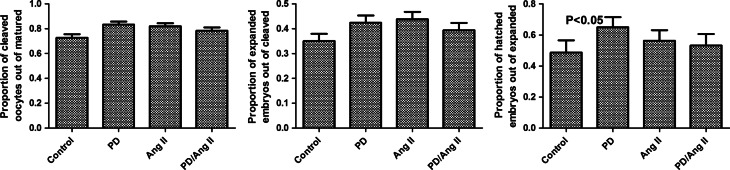
Effect of PD123319 on oocyte cleavage and expansion and hatching of embryos cultured *in vitro*. There was a significant increase in the proportion of hatched embryos in the group treated with PD123319 compared with control (*P*<0.05). All values are presented as mean±s.e.m., *n*=5 cultures.

Treatment with PD123319 was used to investigate the effect of AGTR2 inhibition on embryo development. Approximately 60 oocytes were used for each treatment in each of five experimental replicates. PD123319 had no effect on oocyte cleavage or embryo expansion but it significantly increased (*P*<0.05) the proportion of hatched embryos (Fig. 6).

## Discussion

This study is the first demonstration of mRNA expression and protein localisation for AGTR1 and AGTR2 in the pre-implantation bovine embryo. Under present conditions, there was no detectable expression of *AGT, ACE* or *REN*. The culture of bovine embryos with Ang II and receptor antagonists revealed that blocking of AGTR2 significantly improved embryo hatching.

The presence of mRNA and protein for AGTR1 and AGTR2 in the pre-implantation embryo (Figs 1 and 3) suggests that the embryo is sensitive to Ang II that is present in the early gestational environment. As *AGT*, the Ang II precursor, as well as *REN* and *ACE*, does not appear to be expressed by the embryo at this stage, it is possible that the embryo is responsive to Ang II produced by the mother. REN, AGT and ACE are present in human endometrium and vary cyclically (21); Ang II could therefore be a developmental signal between the mother and the developing embryo.

Immunolocalisation of AGTR1 and AGTR2 (Fig. 3) showed that both the receptors are present in the plasma membrane. These receptors belong to the G protein-coupled receptor family, and the presence of AGTR1 and AGTR2 in the plasma membrane of rat pheochromocytoma cells, rat kidney and many others has been well documented (5, 22). However, AGTR1 was mainly found in granular-like structures in the cytoplasm of trophectoderm cells and inner cell mass, suggesting that AGTR1 had been internalised into the cytoplasm (Fig. 3). Desensitisation and internalisation is one of the mechanisms by which AGTR1 is regulated (23, 24). The observation that the granular-like structures showed a very strong signal for AGTR1 suggests that this receptor is active in the hatched blastocyst.

In this study, AGTR2 protein was seen mainly in the nuclear membrane and the mitotic spindle. In contrast to AGTR1, the AGTR2 is not internalised (24). AGTR2 and AGTR1 have been shown by confocal microscopy to be present in the nuclear membrane of rat ventricular cardiomyocytes and in adult sheep kidney cortex (25, 26). Their presence on the nuclear membrane was associated with the influence of Ang II on the expression of genes such as nuclear factor κ light chain enhancer of activated B cells (NF-κB) (27, 28) and on nitric oxide production (25). AGTR2 is up-regulated in kidney injury in association with inflammation and apoptosis (29). Apoptosis occurs frequently as a tissue regulatory process in the developing embryo, for example as cells with chromosomal or gene abnormalities are eliminated. It is also necessary for implantation. Ang II has a number of different actions depending on the localisation and density of its receptor. Thus, AGTR2 may play a protective role during early development, increasing the chances of survival of the embryo.

In the human placenta, the AGTR2 is mainly localised in cytotrophoblast and extravillous trophoblastic cells (30, 31), and this is in agreement with our observation that *AGTR2* is mainly present in the bovine hatched blastocyst just before implantation on day 19. Trophoblast invasion into the uterus is essential for placental establishment and the initiation of a cascade of events leading to the remodelling of maternal vessels. The involvement of Ang II in implantation has not yet been established. This study focuses on the analysis of RAS components in *in vitro*-produced bovine embryos. However, the availability of elongated trophoectoderm tissue provided an opportunity to extend the investigation towards the implantation period. The presence of AGTR2 around the time of implantation is a new and exciting discovery, but further investigation would be needed to assess its putative role in implantation. In the bovine allantochorionic membrane, AGTR2 is the predominant receptor for Ang II, in contrast to the maternal side, which mainly possesses AGTR1 (10).

Ang II, acting via its main receptors, is known to be involved in a variety of physiological responses in reproductive tissues and in foetuses. Neither of the two Ang II concentrations used in the present experiments (10^−11^ and 10^−9^ M) affected embryo development. The only other available study on (later) embryonic RAS showed that in the presence of different concentrations of Ang II, the best development of organogenesis in the peri-implantation rat embryo occurred at 10^−11^ M Ang II (16); these effects were mediated through the AGTR2s. In bovine (32), it was shown that ACE activity in the endometrium and myometrium was negatively correlated with the duration of gestation. This could indicate a higher Ang II production at the beginning of pregnancy, probably before the embryo starts producing its own Ang II. In agreement with this, ACE activity was positively correlated with the duration of pregnancy in the allantoamniotic membrane. Furthermore, the concentrations of active REN increase at the beginning of bovine pregnancy (33), perhaps indicating increased production of Ang II. Therefore, it is possible that the availability of Ang II changes during pregnancy.

The use of Ang II antagonists, PD123319 and Olmesartan, showed that inhibition of AGTR1 does not affect oocyte cleavage, embryo expansion and embryo hatching. The effect of AGTR1 blockade on pre-implantation embryo development has not been reported previously although studies on *Agtr1a* knockout mice (34) showed that the number of live newborns was significantly reduced in *Agtr1a* deficiency. Histological analyses showed that this was mainly due to placental malformation (34), and absence of *Agtr1a* also caused impaired trophoblast maturation and impaired placental function. However, it is still not clear whether this was due to the absence of *Agtr1a* from the foetal or the maternal side.

Treatment of embryos with the AGTR2 antagonist PD123319 significantly increased the proportion of hatched embryos. There is no previous information on the effect of AGTR2 blockade on pre-implantation embryo development in any species. *Agtr2*-deficient mice are fertile and their offspring develop normally, despite alterations to vascular differentiation in the contractile apparatus. They also show reduced exploratory behaviour and greater stimulation of dipsogenesis after water deprivation, as AGTR2 is involved in neuronal development (35, 36). Tebbs *et al*. (16) showed that PD123319 abolished an Ang II-induced increase in embryonic development (16). AGTR2 is the predominant Ang II receptor in the developing foetus and inhibiting it resulted in reduced somite number and forelimb development (16). A slight decrease in embryo hatching in the presence of the AGTR1 inhibitor and Ang II, although not significant, may suggest that Ang II acts through AGTR2 to lower the number of hatching embryos, but this requires further investigation.

The present results show for the first time that ATG1R and AGTR2 are present in the bovine-hatched blastocyst. As precursor transcripts for Ang II were not detectable, it is possible that the embryo responds to Ang II produced by the mother in the oviduct or endometrium and that this hormone might be involved in specific developmental signalling. It is likely that this communication operates through the AGTR2, as blocking of this receptor was found to increase embryo hatching.

## Author contribution statement

W Pijacka performed the experiments and analyses and drafted the paper. M G Hunter guided the research and assisted in writing the paper. F Broughton Pipkin initiated and guided the research and corrected drafts of the paper. M R Luck reviewed the research and analyses and managed the writing of the paper.

## Figures and Tables

**Table 1:** Primers used for RT-PCR analyses to examine expression of renin–angiotensin system components in bovine embryos.

**Gene name**	**Accession no./ref.**	**Primer sequence** (5′–3′)	**Amplicon size** (bp)	**Annealing temperature** (°C)
*ACTB*	(17)	ACT GGG ACG ACA TGG AGA AGA T	441	55
	TGC AAG TCC AAG GCG ACG T		
*AGT*	ENSBTAT00000016440	TCT CGC TGC TGA GAA GAT CA	384	55
	CAT TCG GGT CAG GAA GTT GT		
*REN*	L43524	GAC CGA GGA CGT CTT CTC C	378	62
	CTT GGA GGC AAA GCC TAC AC		
*ACE*	TC255148	TCT CGC TGC TGA GAA GAT CA	359	60
	CAT TCG GGT CAG GAA GTT GT		
*AGTR1*	NM_174233	CAT TAC GAA TCC CAA AAT TCT ACC	344	60
	AGG CAA TTG TTA AAA TAA GCC AAG		
*AGTR2*	NM_000686	GTG CAA AGT TTT TGG TTC TTT TCT	393	55
		TTC TTC CCA TAG CTA TTC GTC TTC		

## References

[1] Atlas SA (2007). The renin–angiotensin aldosterone system: pathophysiological role and pharmacologic inhibition. Journal of Managed Care Pharmacy.

[2] Nguyen G, Burckle CA, Sraer JD (2004). Renin/prorenin-receptor biochemistry and functional significance. Current Hypertension Reports.

[3] Paul M, Poyan Mehr A, Kreutz R (2006). Physiology of local renin–angiotensin systems. Physiological Reviews.

[4] Morgan L, Broughton Pipkin F, Kalsheker N (1996). Angiotensinogen: molecular biology, biochemistry and physiology. International Journal of Biochemistry & Cell Biology.

[5] de Gasparo M, Catt KJ, Inagami T, Wright JW, Unger T (2000). International union of pharmacology, XXIII. The angiotensin II receptors. Pharmacological Reviews.

[6] Matsubara H (1998). Pathophysiological role of angiotensin II type 2 receptor in cardiovascular and renal diseases. Circulation Research.

[7] Schauser KH, Nielsen AH, Winther H, Dantzer V, Poulsen K (1999). Dominance of type 1 angiotensin II receptor in the nonpregnant and pregnant bovine uterus. Journal of Reproduction and Fertility.

[8] Wijayagunawardane MP, Kodithuwakku SP, DE Silva NT, Miyamoto A (2009). Angiotensin II secretion by the bovine oviduct is stimulated by luteinizing hormone and ovarian steroids. Journal of Reproduction and Development.

[9] Wijayagunawardane MP, Miyamoto A, Taquahashi Y, Acosta TJ, Nishimura M, Sato K (2001). Angiotensin II and atrial natriuretic peptide in the cow oviductal contraction *in vitro*: direct effect and local secretion of prostaglandins, endothelin-1, and angiotensin II. Biology of Reproduction.

[10] Schauser KH, Nielsen AH, Winther H, Dantzer V, Poulsen K (1998). Autoradiographic localization and characterization of angiotensin II receptors in the bovine placenta and fetal membranes. Biology of Reproduction.

[11] Grady EF, Sechi LA, Griffin CA, Schambelan M, Kalinyak JE (1991). Expression of AT2 receptors in the developing rat fetus. Journal of Clinical Investigation.

[12] Schutz S, Le Moullec JM, Corvol P, Gasc JM (1996). Early expression of all the components of the renin–angiotensin-system in human development. American Journal of Pathology.

[13] Zemel S, Millan MA, Aguilera G (1989). Distribution of angiotensin II receptors and renin in the mouse fetus. Endocrinology.

[14] Zemel S, Millan MA, Feuillan P, Aguilera G (1990). Characterization and distribution of angiotensin-II receptors in the primate fetus. Journal of Clinical Endocrinology and Metabolism.

[15] Millan MA, Carvallo P, Izumi S, Zemel S, Catt KJ, Aguilera G (1989). Novel sites of expression of functional angiotensin II receptors in the late gestation fetus. Science.

[16] Tebbs C, Pratten MK, Broughton Pipkin F (1999). Angiotensin II is a growth factor in the peri-implantation rat embryo. Journal of Anatomy.

[17] McDougall K, Beecroft J, Wasnidge C, King WA, Hahnel A (1998). Sequences and expression patterns of alkaline phosphatase isozymes in preattachment bovine embryos and the adult bovine. Molecular Reproduction and Development.

[18] Chanrachakul B, Matharoo-Ball B, Turner A, Robinson G, Broughton-Pipkin F, Arulkumaran S, Khan RN (2003). Reduced expression of immunoreactive β2-adrenergic receptor protein in human myometrium with labor. Journal of Clinical Endocrinology and Metabolism.

[19] Blondin P, Sirard MA (1995). Oocyte and follicular morphology as determining characteristics for developmental competence in bovine oocytes. Molecular Reproduction and Development.

[20] Kelly RD, Alberio R, Campbell KH (2010). A-type lamin dynamics in bovine somatic cell nuclear transfer embryos. Reproduction, Fertility, and Development.

[21] Johnson IR (1980). Renin substrate, active and acid-activatable renin concentrations in human plasma and endometrium during the normal menstrual cycle. British Journal of Obstetrics and Gynaecology.

[22] Kambayashi Y, Takahashi K, Bardhan S, Inagami T (1994). Molecular structure and function of angiotensin type 2 receptor. Kidney International.

[23] Qian H, Pipolo L, Thomas WG (2001). Association of β-arrestin 1 with the type 1A angiotensin II receptor involves phosphorylation of the receptor carboxyl terminus and correlates with receptor internalization. Molecular Endocrinology.

[24] Turu G, Szidonya L, Gaborik Z, Buday L, Spat A, Clark AJ, Hunyady L (2006). Differential β-arrestin binding of AT1 and AT2 angiotensin receptors. FEBS Letters.

[25] Gwathmey TM, Shaltout HA, Pendergrass KD, Pirro NT, Figueroa JP, Rose JC, Diz DI, Chappell MC (2009). Nuclear angiotensin II type 2 (AT2) receptors are functionally linked to nitric oxide production. American Journal of Physiology. Renal Physiology.

[26] Tadevosyan A, Maguy A, Villeneuve LR, Babin J, Bonnefoy A, Allen BG, Nattel S (2010). Nuclear-delimited angiotensin receptor-mediated signaling regulates cardiomyocyte gene expression. Journal of Biological Chemistry.

[27] Ruiz-Ortega M, Lorenzo O, Ruperez M, Konig S, Wittig B, Egido J (2000). Angiotensin II activates nuclear transcription factor κB through AT(1) and AT(2) in vascular smooth muscle cells: molecular mechanisms. Circulation Research.

[28] Wolf G, Wenzel U, Burns KD, Harris RC, Stahl RA, Thaiss F (2002). Angiotensin II activates nuclear transcription factor-κB through AT1 and AT2 receptors. Kidney International.

[29] Ruiz-Ortega M, Esteban V, Suzuki Y, Ruperez M, Mezzano S, Ardiles L, Justo P, Ortiz A, Egido J (2003). Renal expression of angiotensin type 2 (AT2) receptors during kidney damage. Kidney International. Supplement.

[30] Tower CL, Lui S, Charlesworth NR, Smith SD, Aplin JD, Jones RL (2010). Differential expression of angiotensin II type 1 and type 2 receptors at the maternal–fetal interface: potential roles in early placental development. Reproduction.

[31] Williams PJ, Mistry HD, Innes BA, Bulmer JN, Pipkin FB (2010). Expression of AT1R, AT2R and AT4R and their roles in extravillous trophoblast invasion in the human. Placenta.

[32] Schauser KH, Nielsen AH, Dantzer V, Poulsen K (2001). Angiotensin-converting enzyme activity in the bovine uteroplacental unit changes in relation to the cycle and pregnancy. Placenta.

[33] Langer B, Grima M, Coquard C, Bader AM, Schlaeder G, Imbs JL (1998). Plasma active renin, angiotensin I, and angiotensin II during pregnancy and in preeclampsia. Obstetrics and Gynecology.

[34] Walther T, Jank A, Heringer-Walther S, Horn LC, Stepan H (2008). Angiotensin II type 1 receptor has impact on murine placentation. Placenta.

[35] Arce ME, Sanchez SI, Aguilera FL, Seguin LR, Seltzer AM, Ciuffo GM (2011). Purkinje cells express angiotensin II AT(2) receptors at different developmental stages. Neuropeptides.

[36] Hein L, Dzau VJ, Barsh GS (1995). Linkage mapping of the angiotensin AT2 receptor gene (Agtr2) to the mouse X chromosome. Genomics.

